# Four Methods of Recruiting Couples Into a Longitudinal Study of Physical Activity in People With Osteoarthritis: Recruitment, Retention, and Lessons Learned

**DOI:** 10.3389/fpubh.2018.00197

**Published:** 2018-07-18

**Authors:** Dana L. Carthron, Ashley Phillips, Carmen C. Cuthbertson, Katrina R. Ellis, Mary Altpeter, Leigh F. Callahan, Stephanie Bahorski, Christine Rini

**Affiliations:** ^1^College of Nursing, Michigan State University, East Lansing, MI, United States; ^2^Thurston Arthritis Research Center, University of North Carolina, Chapel Hill, NC, United States; ^3^Department of Epidemiology, Gillings School of Global Public Health, University of North Carolina, Chapel Hill, NC, United States; ^4^Department of Health Behavior, Gillings School of Global Public Health, University of North Carolina, Chapel Hill, NC, United States; ^5^Division of Rheumatology, Allergy and Immunology, Department of Medicine, University of North Carolina, Chapel Hill, NC, United States; ^6^Cancer Prevention and Control Program, John Theurer Cancer Center, Hackensack University Medical Center, Hackensack, NJ, United States; ^7^Department of Oncology, School of Medicine, Georgetown University, Washington, DC, United States

**Keywords:** physical activity, arthritis, couples, aging, chronic disease, recruitment, retention

## Abstract

Increases in physical activity can reduce joint pain among people with osteoarthritis (PWOA) who are insufficiently physically active. Because evidence suggests that social support from intimate partners may help PWOA become more active, researchers have been interested in recruiting couples to studies of physical activity interventions; however, little guidance exists describing efficient and effective strategies for engaging couples in research. We describe methods used to recruit couples and contrast methods in terms of the proportion of individuals enrolled, sample demographic composition, retention, and resources. We used four recruitment methods to enroll couples in a longitudinal study of PWOA: (1) visiting community sites, (2) sending university-wide emails, (3) contacting patients identified through electronic medical records (EMR), and (4) partnering with a county-based osteoarthritis (OA) research cohort. We found that these methods differed in their challenges and contribution to enrollment goals but demonstrated similar levels of retention. We contacted 747 PWOA; 56% were screened for eligibility and 23% enrolled in the study. The largest proportion of participants recruited were from the email method (35.1%), followed by the community (26%), EMR (22.0%), and OA cohort (19.6%). Couples enrolled through the different methods differed by age, employment, education, and household income. Across the methods for both PWOA and partners, over 80% of participants were non-Hispanic white, about 11% were non-Hispanic black, and 6–8% identified as another race. Over 12 months of follow-up, 31 (17.9%) PWOA and 36 (20.8%) partners were lost to follow-up. Using four distinct recruitment methods allowed us to meet recruitment goals and provided a broader, more diverse population compared to using one method. We recommend that researchers consider several recruitment methods to meet enrollment goals, to ensure a diverse sample, and to match available resources. The lessons learned from this research fill a critical gap in the understanding of how to overcome barriers to recruiting and retaining couples in behavioral research.

## Introduction

The burden of osteoarthritis (OA) is substantial because of its adverse effects on quality of life, productivity, and healthcare costs (1). The most common form of arthritis, OA affects more than 30 million adults in the United States, and its impact is expected to increase with the rising prevalence of obesity and aging of the population (2, 3). A consensus supports multimodal treatments for OA, with increased physical activity (PA) as a central component (4). Modest increases in PA can reduce OA pain and improve physical functioning, mental health, and quality of life (5). Because only 13% of people with hip or knee OA achieve recommended levels of PA (6), developing effective interventions to help inactive people with OA (PWOA) increase PA is an important public health goal.

Although PA interventions are efficacious for inactive PWOA, their effects may be modest or short-lived (7, 8). There is a pressing need for interventions that address barriers to PA in a way that promotes lasting behavior change capable of improving health and well-being in PWOA. The social context of PA offers valuable insights into how to achieve this goal (9). Intimate partners can help each other make lasting changes in health behaviors such as PA (10). For PWOA, partner support has been linked to increased PA (11, 12). This association is likely due to social, emotional, and resource interdependence within couples (10, 13–15) and the fact that couples are well-positioned to give and receive support for lifestyle change (16, 17).

To learn the best strategies for leveraging partner support to develop more powerful PA interventions, and to conduct research to evaluate or implement these interventions, researchers must overcome challenges of recruiting and retaining couples, which are more complex than challenges involved in engaging individuals in research. It is important to identify efficient and effective recruitment and retention strategies for involving couples in OA intervention research. Unfortunately, few reports describe recruitment and retention rates and strategies for couple-focused behavioral interventions (18). This is a significant barrier to increasing the availability of couple-focused interventions and similar health promotion programs targeting interpersonal-level determinants of health behaviors.

Information that is available about recruiting and retaining couples focus on couples that differ in important ways from those coping with osteoarthritis. For instance, most of these studies (19–23) reported challenges of recruiting couples to studies of cancer populations. Although couples coping with cancer tend to be older adults, and thus similar in age to couples coping with osteoarthritis, recruitment and retention challenges may differ substantially for couples affected by cancer compared to those affected by chronic, debilitating illness such as osteoarthritis. Several other studies (24, 25) have examined recruitment to studies focused on prevention of HIV and other sexually transmitted infections. Differences between the demographic characteristics of these samples and the targeted public health problem reduce the likelihood that findings from these studies would inform a study of older couples coping with osteoarthritis.

This article highlights challenges, successes, and lessons learned regarding recruitment and retention of PWOA and their partners for a couple-focused study that used four distinct recruitment methods, several of which were community based. It has two objectives: (1) to describe four methods used to recruit couples, and (2) to contrast these methods in terms of the proportion of participants that completed the recruitment process, sample demographic composition, retention, and required resources. Our overarching goal is to inform best practices for recruiting partners into couple-focused OA intervention studies and, potentially, other studies of couples by providing concrete recommendations based on practical issues we experienced and lessons we learned.

## Methods

### Study overview

The *Partners in Active Living Study* (PALS) was a one-year longitudinal study of inactive PWOA and their partners. PALS investigated partner support processes and their association with sustained increases in PA among PWOA and their partners. All couples completed a 2$\frac{1}{2}$ h small-group “Active Living” class (ALC) that taught couples about OA, PA, and social support for PA in couples. They were also given a workbook developed as part of an evidence-based lifestyle intervention, *Active Living Every Day* (ALED) (26, 27). PWOA were asked to read workbook chapters and complete activities to incorporate more PA into their daily routines. Partners could choose to complete the workbook, as well. Participants were also given an educational booklet, developed by the study team, describing couple-focused strategies for giving and getting support for PA. In addition to completing self-report measures at an in-person baseline visit, 1 week later, and in the ALC, all participants wore accelerometers to track PA in the week before and after the ALC, and then completed self-report measures and wore accelerometers at four follow-up assessments 1 week (FU1), 3 months (FU2), 6 months (FU3), and 1 year (FU4) following the ALC. This study was approved by and carried out in accordance with the recommendations of the Office of Human Research Ethics Biomedical Institutional Review Board at the University of North Carolina at Chapel Hill (UNC). All subjects gave written informed consent in accordance with the Declaration of Helsinki.

### Participants

Eligible PWOA had symptomatic hip or knee OA diagnosed by a healthcare provider or had probable OA (frequent joint pain, limitation of the hip or knee for at least 6 months, and 50 years or older). In addition, they possessed adequate cognitive functioning [assessed with the Blessed Memory Concentration Test, Katzman et al. (28)] and English proficiency, were insufficiently active (<120 min moderate to vigorous PA per week), were able to walk unaided, had no medical comorbidities that contraindicated PA, had no recent hip or knee surgery, were interested in increasing their PA, and were married or in a marriage-like relationship with a cohabitating partner who was willing to participate in the study. Partners of PWOA had to be English-proficient adults with adequate cognitive function; they may or may not have had OA and may or may not have been interested in increasing PA.

### Recruitment

PALS used four approaches to recruit couples in North Carolina communities near UNC between April 2014 and November 2015: (1) visiting community sites, (2) sending emails to UNC affiliates, (3) contacting patients identified in UNC hospital electronic medical records (EMRs), and (4) partnering with an existing OA longitudinal research cohort. One recruitment method was used to contact participants, although it is possible participants may have learned about the study through multiple methods.

#### Community sites

We recruited couples at three continuing care retirement communities (CCRCs), four public senior centers, and several community events near UNC. We met with CCRC administrators, senior center facility administrators, and CCRC resident research committees to gain approval to recruit at their sites. CCRC representatives announced the study in correspondence to residents and through bulletin board notices. We offered presentations about OA, exercise, and the study to interested groups at the CCRCs. Additionally, recruitment at senior centers was conducted in association with exercise classes, with the assistance of on-site physical therapy staff, and at events such as early voting and health fairs.

#### University mass email

We announced the study to UNC faculty, staff, and students who opted to receive notices through the university mass email system. Emails described the study eligibility criteria, compensation, contact information, institutional review board approval, and study funding. Interested individuals contacted the study team.

#### Electronic medical records

We used the EMRs of the UNC hospital system to identify potential participants who had ICD-9 codes for OA or OA symptoms, were married, and lived within 50 miles of the study site. These individuals were mailed recruitment letters describing the study, and received up to three follow-up phone calls to determine their interest in participating.

#### Johnston county osteoarthritis project

We contacted participants of the Johnston County Osteoarthritis Project (JoCo), a population-based study that has followed participants with and without OA for over 25 years (29). At baseline, JoCo participants included more than 3,000 men and women in a mostly rural North Carolina county with a substantial proportion of residents with low income and low education (30). We approached the JoCo cohort because the PALS Study investigators had longstanding collaborations and partnerships with the JoCo investigators. Additionally, many of the JoCo cohort participants agreed to be contacted for other research studies, which gave us access to a large racially diverse population with OA. Project staff compiled a list of surviving JoCo participants who had agreed to be contacted for additional research projects, had symptoms of hip or knee OA, were aged >50, and were married. A record of all attempted contacts with potential participants was not maintained during the recruitment period, but all potential participants who interacted with study staff were recorded in a database.

### Screening and enrollment

We approached PWOA first, obtained consent for screening, and completed brief screening interviews of approximately 20 min. For those who met eligibility criteria and allowed us to approach their partners, we obtained consent for screening from partners, and completed brief screening interviews (10 min). Most screening interviews were conducted by phone. A couple was considered enrolled after providing consent at the baseline visit, after which they received baseline questionnaires, accelerometers, and instructions.

### Retention

We used various methods to enhance retention in the study's five assessments completed over the 12 months following the baseline visit. These methods included making reminder phone calls for study activities, building rapport with participants, and providing monetary incentives for completed activities.

#### Reminder calls

Study personnel conducted reminder phone calls 2 days before baseline appointments and 2 days before the ALC. Two days before each follow-up assessment (FU1-FU4), staff contacted participants to confirm receipt of materials and to instruct them to wear accelerometers for 1 week, complete questionnaires, and return materials by mail. When necessary, staff contacted participants regarding late or missing materials and answered participants' questions about the study.

#### Rapport-building

Building rapport with participants was expected to maintain interest and increase mutual trust. When practical, the same study team member served as the primary contact for a couple throughout the study. Staff communicated their willingness to accommodate participants' schedules by reviewing procedures with each participant upon enrollment and by offering flexibility when scheduling study activities. Staff took care to communicate with and demonstrate interest in each member of the couple to ensure each felt appreciated for contributing time and effort. For example, each individual received a separate thank-you letter and check at each time point.

#### Incentives

Each participant earned $40 after attending an ALC and submitting their baseline assessment; $20 after returning materials for FU1, FU2, and FU3; and $40 for returning materials for FU4. Thus, incentives for study completion potentially totaled $140 per individual. Additionally, participants received tote bags, pens, refreshments, brochures about OA and PA, and workbooks at the ALC.

### Statistical methods

We examined the following outcomes for each recruitment method: proportion of participants who completed each recruitment step, demographic characteristics of enrolled participants, retention, missing follow-up data, and completion of the ALED workbook. Demographic characteristics included age, gender, education (<college, 4-year college, graduate degree), ethnicity/race (non-Hispanic white, non-Hispanic black, other), and employment (employed, retired, other; participants were allowed to give multiple answers to employment status).We further summarized characteristics of recruitment methods by considering staff time, material costs, and the skills, resources, and access required to reach potential participants.

To examine differences across the four recruitment methods, we used Pearson chi-square and Fisher exact tests for categorical variables and one-way analysis of variance (ANOVA) for continuous variables. For significant results (*p* < 0.05), we examined pairwise comparisons to determine which subgroups differed. We used the Tukey-Kramer method for pairwise comparisons of continuous variables and post-hoc chi-square testing with Bonferroni adjustment for categorical variables. Analyses were conducted with SAS Version 9.4.

## Results

### Recruitment

Each recruitment method produced challenges for evaluating the number of potentially eligible people exposed to study information. For community recruitment, we could not track the number of people who learned of our study but did not contact staff. By email, we announced the study to approximately 7,000 UNC faculty, staff, and students, though we were unable to determine if emails were read. Emails reached a broad audience, many of whom were likely ineligible (i.e., people without OA). For EMR recruitment, we mailed recruitment letters to approximately 2,115 patients with strong potential to be eligible according to medical records. However, we could not determine how many letters reached or were read by the addressees. Although we attempted to phone patients, we cannot know whether those we were unable to contact were uninterested or unreachable for other reasons. For the JoCo cohort, we were able to track all potential participants who interacted with study staff and their status.

Over the 19-month recruitment period, staff made contact with 747 potential PWOA participants; more than half (56.1%) consented to screening (Table 1). The proportion who consented to screening was highest for email and lowest for JoCo recruitment (67.5%, 50.2% respectively). The highest proportion of eligible PWOA was recruited by email compared to EMR (46.8 vs. 29.3%, *p* = 0.0030) and JoCo (24.7%, *p* = 0.0003). Among PWOA contacted for recruitment, the two most common reasons they were not eligible were: (1) not meeting symptomatic OA criteria (28.7%) or (2) engaging in >120 min per week of moderate to vigorous PA (40.0%) (Supplementary Table 1).

**Table 1:** Number and proportion of couples that completed study recruitment steps in PALS by recruitment method.

	**Community**		**Email**		**EMR**		**JoCo**		**Total**		
**Recruitment step**	***n***	**%**	***n***	**%**	***n***	**%**	***n***	**%**	***n***	**%**	***p***
PWOA contact with staff	96	100.0	77	100.0	355	100.0	219	100.0	747	100.0	
PWOA screened	54	56.3	52	67.5	203	57.2	110	50.2	419	56.1	0.062
PWOA eligible^a^^,^^b^	30	31.3	36	46.8	104	29.3	54	24.7	224	30.0	0.004
Partners screened^a^^,^^b^	26	27.1	34	44.2	99	27.9	50	22.8	209	28.0	0.005
Partners eligible^a^^,^^b^	26	27.1	34	44.2	98	27.6	48	21.9	206	27.6	0.003
Couples enrolled^a^^,^^b^	25	26.0	27	35.1	78	22.0	43	19.6	173	23.2	0.039

Reported p-values represent the overall comparison of four recruitment methods by Pearson chi-square tests. Significant post hoc chi-square differences (Bonferroni corrected p = 0.0083 for 6 pairwise comparisons) are indicated by superscript ^a−b^. PALS, Partners in Active Living Study; EMR, electronic medical record; JoCo, Johnston County Osteoarthritis Project; PWOA, people with osteoarthritis.

a *Email vs. EMR significantly different*.

b *Email vs. JoCo significantly different*.

Across the four recruitment methods, nearly all partners of eligible PWOA consented to screening and met eligibility criteria. Because the highest proportion of eligible PWOA were recruited by email, the highest proportion of partners completing these steps was likewise recruited by this method (screened 44.2%, eligible 44.2%). This proportion was higher than the proportion of partners screened and found eligible from EMRs (screened 27.9%, *p* = 0.0051; eligible 27.6%, *p* = 0.0043) and JoCo (screened 22.8%, *p* = 0.0004; eligible 21.9%, *p* = 0.0002) methods.

Of 747 potential participants contacted across all methods, 173 couples (23.2%) enrolled in the study. The largest proportion of enrolled couples was recruited by email (35.1%), followed by the community (26%) and from EMRs (22.0%). The smallest proportion was recruited from JoCo (19.6%).

### Demographic characteristics of enrolled couples

Couples enrolled through the four methods differed by age, employment status, education, and household income (Table 2). PWOA recruited by email were younger (*M*_age_ = 57.8 years) than PWOA recruited by the other three methods (*M*_age_ ranged from 65.0 to 69.1 years, *p value*s ranged from 0.0138 to <0.0001). PWOA recruited by the email and community methods were more likely to be employed (76.9 and 58.3%, respectively) than PWOA recruited from JoCo (23.3%) (*p* = 0.0041 and <0.0001, respectively), and PWOA recruited through email were more likely to be employed than those recruited through EMRs (33.8%; *p* = 0.0001) In contrast, PWOA recruited by EMR and from JoCo were more likely to report being retired (58.4 and 72.1%) compared to PWOA recruited by email (15.4%) (*p* = 0.0001 and <0.0001, respectively). Those recruited from the community did not differ from others in their likelihood of being retired. PWOA recruited from JoCo reported lower levels of education (*p* < 0.0001 for each pairwise comparison of JoCo participants vs. each) and household income (JoCo vs. community *p* = 0.0004, vs. email or vs. EMR < 0.0001) than PWOA recruited from the other three methods. We found no differences in the gender or race of PWOA across recruitment methods. Overall, most PWOA were female (65.3%) and most self-identified as non-Hispanic white (82.9%) with 11.2% identifying as non-Hispanic black.

**Table 2:** Demographic characteristics of PWOA and partner participants by recruitment method.

	**Community**		**Email**		**EMR**		**JoCo**		**Total**		
	***n*** = **25**		***n*** = **27**		***n*** = **78**		***n*** = **43**		***n*** = **173**		
**Characteristic**	***n***	**% or M *(SD)***	***n***	**% or M *(SD)***	***n***	**% or M *(SD)***	***n***	**% or M *(SD)***	***n***	**% or M *(SD)***	***p***
**PWOA**											
Age^a^^,^^b^^,^^c^		65.0 (8.7)		57.8 (9.5)		66.1 (8.6)		69.1 (6.3)		65.4 (8.9)	<0.001
Female	17	70.8	16	61.5	48	62.3	30	69.8	111	65.3	0.762
Education ^c^^,^^d^^,^^e^											<0.001
<college	4	16.7	7	26.9	24	31.2	35	81.4	70	41.2	
4-year college	8	33.3	7	26.9	19	24.7	7	16.3	41	24.1	
Graduate	12	50.0	12	46.2	34	44.2	1	2.3	59	34.7	
Ethnicity/Race											0.322
NH White	20	83.3	22	84.6	68	88.3	31	72.1	141	82.9	
NH Black	3	12.5	2	7.7	5	6.5	9	20.9	19	11.2	
Other^f^	1	4.2	2	7.7	4	5.2	3	7.0	10	5.9	
Employment^g^											
Employed^b^^,^^c^^,^^d^	14	58.3	20	76.9	26	33.8	10	23.3	70	41.2	<0.001
Retired ^b^^,^^c^	10	41.7	4	15.4	45	58.4	31	72.1	90	52.9	<0.001
Other^h^	5	20.8	1	3.9	12	15.6	8	18.6	26	15.3	0.269
Household income^c^^,^^d^^,^^e^											
<45,000	2	10.0	2	8.3	6	8.6	23	56.1	33	21.3	<0.001
45,000–89,999	6	30.0	6	25.0	23	32.9	13	31.7	48	31.0	
90,000–119,999	3	15.0	10	41.7	18	25.7	2	4.9	33	21.3	
>120,000	9	45.0	6	25.0	23	32.9	3	7.3	41	26.5	
**PARTNERS**											
Age^b^^,^^c^		64.5 (8.9)		58.5 (9.1)		66.8 (10.2)		69.4 (7.2)		65.9 (9.7)	<0.001
Female	8	33.3	10	38.5	30	39.0	13	30.2	61	35.9	0.788
Education^c^^,^^d^^,^^e^											<0.001
<college	7	29.2	9	34.6	23	30.3	37	88.1	76	45.2	
4-year college	5	20.8	7	26.9	21	27.6	3	7.1	36	21.4	
Graduate	12	50.0	10	38.5	32	42.1	2	4.8	56	33.3	
Ethnicity/Race											0.276
NH White	18	75.0	22	84.6	65	85.5	33	76.7	138	81.7	
NH Black	3	12.5	3	11.5	4	5.3	8	18.6	18	10.7	
Other^f^	3	12.5	1	3.9	7	9.2	2	4.7	13	7.7	
Employment^g^											
Employed^b^^,^^c^	13	54.2	21	80.8	30	39.5	11	25.6	75	44.4	<0.001
Retired^b^^,^^c^	10	41.7	6	23.1	41	54.0	31	72.1	88	52.1	0.001
Other^h^	4	16.7	3	11.5	16	21.1	3	7.0	26	15.4	0.211

Reported p-values represent overall comparison of four recruitment methods by Pearson chi-square tests and one-way ANOVA as appropriate. Superscript ^a−e^ indicates significant pairwise comparisons for continuous variables conducted using Tukey-Kramer test and significant post hoc chi-square differences for categorical variables (Bonferroni corrected p = 0.0083). EMR = electronic medical record, JoCo = Johnston County Osteoarthritis Project, NH = non-Hispanic, PWOA = people with osteoarthritis.

a *Community vs. Email significantly different*.

b *Email vs. EMR significantly different*.

c *Email vs. JoCo significantly different*.

d *Community vs. JoCo significantly different*.

e *EMR vs. JoCo significantly different*.

f *Other ethnicity/race includes Hispanic, Asian/Pacific Islander, American Indian/Alaska Native, and multiracial*.

g *Participants can give multiple answers*.

h *Other employment status includes employed but unable to work due to illness or disability, employed but on medical or family leave, and doing unpaid or voluntary work*.

Demographic characteristics of partner participants were very similar to the PWOA characteristics, except most partners were male (64.1%). We found similar trends for partner participants as previously described for PWOA with regard to differences in demographic characteristics by recruitment method (Table 2).

### Retention and missing assessments

**Figure 1:**
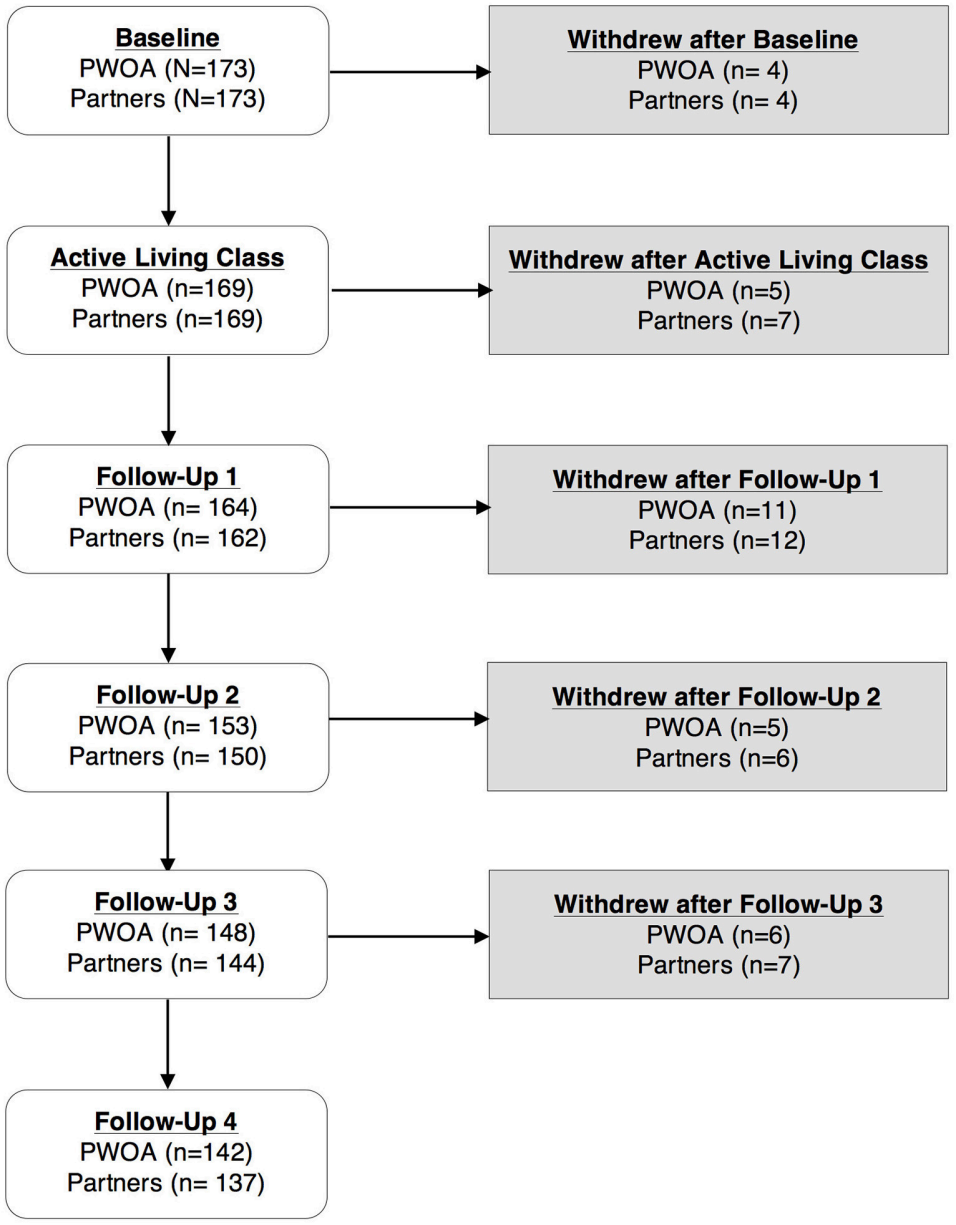
Retention of PWOA and partners in the Partners in Active Living Study. PWOA, people with osteoarthritis; follow-up 1 = 1 week after ALC; follow-up 2 = 3 month after ALC; follow-up 3 = 6 month ALC; follow-up 4 = 12 month after ALC.

Over the 12-month study, 31 (17.9%) PWOA and 36 (20.8%) partners were lost to follow-up (Figure 1). We observed no significant differences in attrition across recruitment methods, though rates ranged from just over 29% for couples recruited by email (PWOA 29.6%, partner 29.6%) to 18% or less for couples recruited by EMR (PWOA 14.1%, partner 18.0%). The largest attrition rate occurred between FU 1 and FU 2, when 11 (6.4%) PWOA and 12 (6.9%) partner participants were lost. At other follow-up time points, attrition ranged from 2.3 to 3.5%. Most participants (PWOA 89.4%, partner 92%) returned all questionnaires over the 12-month follow-up period with no significant differences by recruitment method. The follow-up assessment with the largest nonresponse rate was FU 2 (6.5% of PWOA, 6.0% of partner participants). Otherwise, nonresponse ranged from 0 to 4.1%. Reduced attrition and nonresponse may be attributed to the fact that we implemented further retention strategies after noting high attrition at FU2. A large proportion of PWOA reported completing any of the ALED workbook (ranged from 65 to 94%) and fewer partners reported completing any of the workbook (ranged from 45 to 74%). No differences were detected by site in completing the ALED workbook.

### Comparison of resources required among recruitment methods

In addition to differences in the quantity and characteristics of participants recruited and retained, logistical differences were also observed across recruitment methods (Table 3). Relative to the number of participants ultimately enrolled, community recruitment required the greatest amount of staff effort, whereas email recruitment required the least. For example, for the community recruitment, the principal investigators spent much time discussing the study with administrators at the three CCRCs and four community centers to see if it was possible to recruit at their locations. Investigators also spent time preparing and submitting a description of the study to research committees of each site for their review. After the sites agreed to the recruitment, investigators and study staff conducted 1-h presentations about the study at each location. At the senior centers staff were on site for about 4 h each to do in-person screening. Additionally, staff spent time at multiple community events to try to recruit participants. Staff travel was involved for going to the CCRCs, senior centers, and the community events. In contrast, the other three recruitment methods did not require as much staff effort. The EMR method required staff to query the EMR database, to prepare and mail letters to 2,115 potential participants, and to call potential participants. Due to existing partnerships with the JoCo study, staff effort was mainly spent contacting all participants directly over the phone to determine their interest in the study. In our experience the email method required the least staff effort of all methods, as potential participants interested in the study contacted study staff.

**Table 3:** Summary of resources for recruitment methods employed in PALS.

	**Community**	**Email**	**EMR**	**JoCo**
**RECRUITMENT TASKS THAT REQUIRED STAFF TIME**				
Gain IRB approval (UNC)	X	X	X	X
Talk with contacts at each site	X			X
Prepare materials to gain approval from the sites	X			
Prepare for the on-site presentations	X			
In-person presentations	X			
In-person recruitment for sites	X			
In-person recruitment for community events	X			
Travel time for sites and community events	X			
Query and develop list of potential participants			X	
Prepare letters for EMR potential participants			X	
PWOA contact with staff by phone	X	X	X	X
PWOA screened by phone	X	X	X	X
Partners screened by phone	X	X	X	X
**MATERIALS**				
Brochures, flyers	X			
Personalized letters			X	
**ACCESSIBILITY**^a^				
UNC institutional approval	X	X	X	X
Approval from gatekeepers at each site	X			X
**SKILLS AND RESOURCES REQUIRED**				
In-person recruitment	X			
Travel	X			
Public speaking	X			
Query EMR database			X	
Computer	X	X	X	X
Phone	X	X	X	X

PALS, Partners in Active Living Study; EMR, electronic medical record; JoCo, Johnston County Osteoarthritis Project.

a *Accessibility of reaching potential participants*.

Materials incurred during recruitment involved printing brochures, flyers, screening materials, and recruitment letters. Brochures and flyers were used with the community method and were posted at each recruitment site. Personalized letters were used for the EMR method, which included supplies such as paper, envelopes, mailing labels, return address labels, and postage. Email recruitment required the least materials as this method only required sending an email.

The accessibility of recruitment methods also varied. The use of the JoCo database to recruit participants required not only the availability of the cohort, but approval from and coordination with the JoCo study principal investigator. Using EMRs was possible because of our affiliated medical center, but required IRB approval and a formal data request. Similarly, the use of a university mass email system was limited to university affiliates and required institutional approval. Community recruitment often required approval of official or unofficial gatekeepers, but generally involved fewer formal barriers to access and is likely to be more easily available to researchers at various types of institutions. Finally, the skills and resources required varied across recruitment methods. Community recruitment was unique in its need for travel and in-person contact.

## Discussion

During the planning stages of our study, we found limited information regarding best practices for recruiting and retaining older couples in research studies and no information specific to recruiting couples into OA studies. However, employing four distinct recruitment approaches allowed us to meet enrollment goals while providing an opportunity to compare approaches. Given some differences in the characteristics of participants recruited, using four recruitment methods provided us with a sample that represented a broader, more diverse cross-section of our population of interest than using one recruitment method would have offered.

### Lessons learned across methods

#### Partner recruitment and retention

After PWOA expressed interest and were determined to be eligible, we recruited most partners. Limited partner eligibility criteria likely contributed to the high proportion of eligible partners. Similar numbers of PWOA and partners continued to participate throughout this longitudinal study, suggesting that a couple-focused approach can maintain participation even among participants who do not experience the condition of interest. Efforts to ensure both members of the couple felt important to the study may also have contributed to retention of PWOA and partners.

#### Recruitment of underrepresented minorities

Limited racial and ethnic diversity presented a challenge during recruitment and limits how our sample can represent our priority population. A disproportionate number of minorities, particularly African Americans, are affected by knee OA (31). Staff approached an African American church network to enhance recruitment because many researchers have used this strategy. Unfortunately, we were unsuccessful in partnering with this network. The limited diversity among study staff served may have been a barrier. Also, community recruitment is best accomplished by building on established relationships with input and buy-in from community stakeholders (32). Although we had such relationships with some community partners, we had not established them with the church network. Moreover, past research has found that church-based recruitment of African Americans is more successful when recruiters are native to the community and involved with community churches beyond research activities (33). Therefore, we recommend using community-based participatory research methods to build relationships and ensuring that recruitment staff reflect the diversity of the priority population.

### Method-specific lessons learned in recruitment and retention

#### Community sites

Recruitment from local sites was more labor-intensive than other approaches due to the number of onsite meetings necessary to build partnerships and obtain access to potential participants. Furthermore, additional staff time was incurred to create tailored presentations. To combat the challenge of low enrollment from community sites, we widened the geographic scope of the study to include two counties not initially considered. This method yielded limited overall success in recruitment in proportion to the level of staff and material resources required.

#### University mass email

The use of a mass email tool presented unique challenges and benefits. A major limitation of this method was its exclusion of campus community members who did not opt to receive mass email messages. After sending email messages, staff spent time fielding inquiries via email and phone. This method yielded overall success in recruitment, especially in proportion to the limited effort required. Including general eligibility criteria and the study description in emails allowed recipients to consider if the study suited them without direct staff contact.

#### Electronic medical records

The use of EMRs allowed staff to easily identify potentially eligible individuals by filtering data with unique inclusion criteria. Even so, seeking approval to access protected health information and learning to use a complex database required additional staff time. Furthermore, because there had been no prior contact by study personnel, we had to engage and educate potential participants about the study and how they had been identified for recruitment. This method reached a large number of potential participants and resulted in similar recruitment outcomes as the other methods.

#### Johnston county osteoarthritis research project

Working with an existing county-based research cohort had the potential to be an efficient recruitment strategy; it allowed study personnel to quickly identify individuals who were likely to meet inclusion criteria and had already demonstrated willingness to participate in research. However, only individuals who consented *a priori* to be contacted about future research studies were contacted, raising concerns about the generalizability of findings to non-volunteer populations. In addition, a greater-than-expected proportion of potential participants from JoCo were not interested in our study, and many did not meet our complex eligibility criteria.

## Conclusion

Conducting couple-focused research can pose an array of challenges. However, we found it possible to recruit and retain this population for longitudinal research. We recommend that researchers consider several recruitment methods to meet enrollment goals, to ensure a diverse sample, and to match available resources. We have provided information to guide selection of recruitment methods for studies targeting couples that are based on very practical logistical issues. University mass emails were found to be the most effective for our study. Because email respondents assessed their potential eligibility prior to contacting staff, less staff and financial resources were required compared to other methods. However, we also note that combining all recruitment methods was necessary to meet our enrollment goal. Additionally, investigators must be mindful of the community in which they plan to recruit to ensure diversity among study participants. The lessons we learned help fill a critical gap in the understanding of methods for recruiting couples and delineate implications for future work to overcome barriers to recruiting and retaining couples in behavioral research.

## Author contributions

Recruitment and data collection for this study were completed under the supervision of CR. DC, AP, CC, KE, MA, LC, SB, and CR conceived of this paper. DC, AP, and CC drafted the paper with major section contributions and editing of the manuscript from KE, MA, LC, SB, and CR. All authors approved the final version of this manuscript.

### Conflict of interest statement

The authors declare that the research was conducted in the absence of any commercial or financial relationships that could be construed as a potential conflict of interest.
