# Supplementary material for: Four Methods of Recruiting Couples Into a Longitudinal Study of Physical Activity in People With Osteoarthritis: Recruitment, Retention, and Lessons Learned
Source: Front Public Health. 2018 Jul 18;6:197. doi: 10.3389/fpubh.2018.00197 (PMC6058039; doi:10.3389/fpubh.2018.00197)
Supplement: Supplementary file 1 [file Table_1.DOCX]

Supplementary Material

**Four Methods of Recruiting Couples into a Longitudinal Study of Physical Activity in People with Osteoarthritis: Recruitment, Retention, and Lessons Learned**

Dana L. Carthron^*^, Ashley Phillips, Carmen C. Cuthbertson, Katrina Ellis, Mary Altpeter, Leigh F. Callahan, Stephanie Bahorski, Christine Rini

*** Correspondance**:

Dr. Dana L. Carthron

[cartdana@umich.edu](mailto:cartdana@umich.edu)

**Supplementary Table 1.**

*Reasons PWOA Were Not Eligible for PALS as Determined by the Screening Interview*

|  | Community | |  | Email | |  | EMR | |  | JoCo | |  | Total | |
| --- | --- | --- | --- | --- | --- | --- | --- | --- | --- | --- | --- | --- | --- | --- |
|  | *n* = 24 | |  | *n* = 16 | |  | *n* = 99 | |  | *n* = 56 | |  | *n* = 195 | |
| Reason for ineligibility | *n* | % |  | *n* | % |  | *n* | % |  | *n* | % |  | *n* | % |
| **Did not meet symptomatic OA criteria** | 5 | 20.8 |  | 8 | 50.0 |  | 18 | 18.2 |  | 25 | 44.6 |  | 56 | 28.7 |
| Reported OA diagnosis but no frequent pain^a^ or no limitation | 1 | 4.2 |  | 1 | 6.3 |  | 10 | 10.1 |  | 5 | 8.9 |  | 17 | 8.7 |
| No reported OA diagnosis by healthcare provider |  |  |  |  |  |  |  |  |  |  |  |  |  |  |
| And no frequent pain^a^ or no limitation | 3 | 12.5 |  | 3 | 18.8 |  | 8 | 8.1 |  | 20 | 35.7 |  | 34 | 17.4 |
| And report frequent pain^a^ but < 6 months | 1 | 4.2 |  | 0 | 0 |  | 0 | 0 |  | 0 | 0 |  | 1 | 0.5 |
| And report frequent pain^a^ and limitation but age < 50 years | 0 | 0 |  | 4 | 25.0 |  | 0 | 0 |  | 0 | 0 |  | 4 | 2.1 |
| **Engaged in** > **120 minutes of PA per week** | 12 | 50.0 |  | 5 | 31.3 |  | 49 | 49.5 |  | 12 | 21.4 |  | 78 | 40.0 |
| **Had medical condition contraindicating PA** | 4 | 16.7 |  | 3 | 18.8 |  | 18 | 18.2 |  | 10 | 17.9 |  | 35 | 18.0 |
| **Other** | 3 | 12.5 |  | 0 | 0 |  | 14 | 14.5 |  | 9 | 16.1 |  | 26 | 13.3 |
| PWOA denied partner contact by staff | 0 | 0 |  | 0 | 0 |  | 1 | 1.0 |  | 0 | 0.0 |  | 1 | 0.5 |
| No interest in increasing PA | 0 | 0 |  | 0 | 0 |  | 3 | 3.0 |  | 4 | 7.1 |  | 7 | 3.6 |
| Needed assistance walking | 2 | 8.3 |  | 0 | 0 |  | 5 | 5.1 |  | 2 | 3.6 |  | 9 | 4.6 |
| Cognitive impairment | 1 | 4.2 |  | 0 | 0 |  | 1 | 1.0 |  | 0 | 0 |  | 2 | 1.0 |
| Recent hip or knee surgery | 0 | 0 |  | 0 | 0 |  | 1 | 1.0 |  | 2 | 3.6 |  | 3 | 1.5 |
| Unknown | 0 | 0 |  | 0 | 0 |  | 3 | 3.0 |  | 1 | 1.8 |  | 4 | 2.1 |

*Note.* Overall *p* = 0.002 for testing differences of three categories of ineligibility (did not meet symptomatic OA criteria, engaged in > 120 minutes of PA per week, and all other reasons) across recruitment methods. Significant post hoc chi-square differences using a corrected *p* value of 0.0083 conducted with 3 categories of ineligibility confirmed for EMR vs. JoCo. With the exception of “other” reasons, categories of ineligibility are listed in order of appearance on screening questionnaire. Screening did not advance after a respondent was determined ineligible in one category. PWOA=people with osteoarthritis, PALS=Partners in Active Living Study; EMR=electronic medical record, JoCo= Johnston County Osteoarthritis Project, OA=osteoarthritis, PA=physical activity.

^a^Frequent pain is described as pain, aching, or stiffness in a hip or knee joint on most days of the week. ^b^Medical conditions include chest pain, dizziness or recent loss of consciousness, uncontrolled heart disease, certain cancer diagnoses, ongoing cancer therapy, uncontrolled respiratory disease, uncontrolled cerebrovascular disease, recent stroke, and requirement of medical supervision for PA.
